# Development, Normative Data, and Psychometric Properties of the Turkish Semantic Association Test (SEMAT) for Middle‐Aged and Older Adults

**DOI:** 10.1002/brb3.71833

**Published:** 2026-10-07

**Authors:** Samet Tosun, Fenise Selin Karalı, Joël Macoir

**Affiliations:** ^1^ Department of Speech and Language Therapy Biruni University Istanbul Turkey; ^2^ École Des Sciences de La Réadaptation, Faculté de Médecine Université Laval Québec Canada; ^3^ CERVO Brain Research Centre Québec Canada

**Keywords:** aging, semantic association, semantic cognition, semantic memory

## Abstract

**Purpose:**

Semantic associative processing is essential for language, cognition, and communication and is frequently impaired in neurodegenerative disorders. However, no brief Turkish instrument currently assesses semantic associations across both abstract and concrete conceptual domains. This study aimed to develop the Turkish Semantic Association Test (SEMAT), establish normative data, and examine selected psychometric properties.

**Method:**

The SEMAT was developed using stimuli representing abstract, biological, and manmade concepts. Face validity was evaluated through expert review and pilot testing. Normative data were obtained from 365 cognitively healthy Turkish‐speaking adults aged 53–82 years. Demographic effects were examined using multiple regression analyses, with age, education, and sex entered simultaneously as predictors. Given the bounded, negatively skewed score distributions and substantial ceiling effects, empirical percentile‐based norms were generated. Internal consistency and convergent validity were assessed using semantic measures from the Batterie d’Évaluation Cognitive du Langage (BECLA‐Tr). Item‐level difficulty and discrimination were also examined.

**Findings:**

Educational attainment was the only demographic variable independently associated with performance across all SEMAT scores, supporting education‐adjusted norms. The SEMAT demonstrated excellent internal consistency (Cronbach's α = 0.88; McDonald's ω = 0.92). Significant correlations were observed between SEMAT scores and the BECLA‐Tr semantic composite (ρ = 0.41–0.54, all p < 0.001), supporting convergent validity. Item‐level analyses showed generally high accuracy and positive but variable discrimination across items.

**Conclusion:**

These findings indicate that the SEMAT is a brief, culturally appropriate, and psychometrically sound measure of semantic associative processing with preliminary evidence of reliability and convergent validity and with education‐adjusted normative reference data for cognitively healthy middle‐aged and older Turkish‐speaking adults.

## Graphical abstract

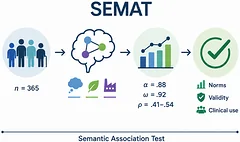

## 1 Introduction

Semantic memory is the system of conceptual knowledge enabling people to recognize and use knowledge about words, objects, persons, facts, and their meanings independently of personal experience (Patterson et al., 2007; Tulving, 1972). It includes knowledge about categories, attributes, functions and relationships between concepts enabling the recognition of objects and events, the generation of inferences, and a wide range of language, cognitive, and communicative activities (Vigliocco and Vinson, 2007; Thye et al., 2024). Semantic memory supports language comprehension and production, reading, reasoning, problem solving, and everyday decision‐making (Binder and Desai, 2011; Lambon Ralph et al., 2017). The integrity of semantic memory is essential given its fundamental role in cognition and communication.

Although semantic knowledge is generally thought to be relatively resistant to the effects of healthy aging compared to other cognitive systems, such as episodic memory (Brickman and Stern, 2009), specific semantic processes have been reported to be vulnerable to age‐related change (Verhaeghen and Poncelet, 2013). Older adults may experience subtle problems with lexical retrieval, slower access to low‐frequency concepts, and slower processing of semantic associations whilst generally maintaining their stores of semantic knowledge (Higby et al., 2019; Verhaeghen and Poncelet, 2013). The results suggest that semantic representations are largely preserved in normal aging, but the mechanisms that support efficient retrieval and manipulation of semantic knowledge may become increasingly compromised with advancing age (Lambon Ralph et al., 2017; Spaan, 2015).

Semantic deficits are found in a broad spectrum of neurological conditions and are a conspicuous characteristic of a number of neurodegenerative diseases. Progressive deterioration of semantic knowledge and semantic associative processing has been documented in individuals with Alzheimer's disease (AD) (Luzzatti et al., 2020; Macoir et al., 2024), mild cognitive impairment (MCI) (Korthauer et al., 2020; Invernizzi et al., 2023), and primary progressive aphasia (PPA), in particular the semantic variant of PPA, in which semantic memory impairment represents a core diagnostic characteristic (Gorno‐Tempini et al., 2011; Hodges & Patterson, 2007). Language‐related deficits, such as impaired naming, reduced verbal fluency, and lexical‐semantic deficits, are among the most common changes in neurodegenerative diseases and may even occur in the prodromal phases of cognitive decline (Adlam et al., 2006; Corbett et al., 2012). Crucially, semantic deficits are linked to reduced communicative effectiveness, diminished functional independence, and poorer quality of life (Croot et al., 2009). As such, clinical evaluation of semantic processing abilities is of utmost importance (Venneri et al., 2018). Therefore, the availability of sensitive and clinically practical instruments able to evaluate semantic associative processing has gained increasing importance both in research and clinical settings (Invernizzi et al., 2023).

One important aspect of semantic memory has to do with distinguishing between concrete and abstract concepts. Concrete concepts (e.g., animals, foods, or common objects) are generally grounded in perceptual and sensory experiences and are associated with visual, auditory, tactile, and motor representations (Martin & Chao, 2001; Villani et al., 2024). On the other hand, abstract concepts are less directly linked to sensory experience and are more reliant on linguistic, contextual, emotional, and relational information for their representation and processing (Kousta et al., 2011; Vigliocco et al., 2014). Such notions include emotions, personality traits, mental states, social values, etc. Contemporary theories of semantic cognition suggest that abstract and concrete concepts differ in the content of their representations and the cognitive and neural mechanisms underlying their processing (Mancano and Papagno, 2023). Concrete concepts seem to activate richer perceptual representations, whereas abstract concepts involve greater executive control, contextual integration, and semantic retrieval processes (Crutch and Warrington, 2005; Lambon Ralph et al., 2017). Neuroimaging and neuropsychological data also support the idea of partly different neural substrates of abstract and concrete semantic processing and show that these types of concepts rely on overlapping but not identical semantic networks (Binder et al., 2009; Mkrtychian et al., 2019; Wang et al., 2010). Importantly, new evidence suggests that different semantic categories may be differentially vulnerable in aging and neurodegenerative disorders (Cousins et al., 2016; Mancano and Papagno, 2023; Cosgrove et al., 2026), rendering the assessment of both abstract and concrete semantic representations particularly relevant for understanding semantic functioning across the lifespan and in clinical populations.

Several instruments have been developed to assess semantic knowledge more directly, such as semantic association tasks like the Pyramids and Palm Trees Test (Howard and Patterson, 1992), which has also been adapted and standardized for the Turkish population (Bozdemir and Gürvit, 2022). The PPTT is an important measure of semantic association abilities, but it primarily involves concrete concepts and object‐based semantic relationships. Furthermore, the PPTT was originally designed to assess semantic access rather than to compare semantic processing across different conceptual domains. Consequently, it provides limited opportunities to examine potential differences between abstract and concrete semantic representations. Likewise, many of the existing semantic measures tend to focus mainly on concrete semantic knowledge, leaving few opportunities for investigating abstract semantic representations. Furthermore, many semantic tests take a long time to administer or are part of larger neuropsychological batteries, limiting their usefulness in both clinical and research settings.

In the Turkish literature, parts of instruments such as the Detection Test for Language Impairments in Adults and the Aged–Turkish Version (Karalı et al., 2025; Tosun et al., 2026a) and the Turkish adaptation of the Batterie d’Évaluation Cognitive du Langage (Karali et al., 2026; Tosun et al., 2026b) include tasks targeting semantic abilities. However, semantic processing is tested with a relatively limited number of items or as part of a broader language battery. None of these tools were specifically designed to test semantic associative processing in both abstract and concrete conceptual domains within a short and targeted assessment framework. These limitations emphasize the need for a clinically practical and psychometrically sound instrument developed specifically for assessing semantic associative processing in Turkish‐speaking adults and older adults.

One potentially useful avenue to assess semantic processing is via semantic association paradigms (Caputi et al., 2016; Reilly et al., 2016). Compared to naming or verbal fluency measures, semantic association measures involve the recognition of relationships between concepts between stimuli, with relatively limited demands on lexical retrieval, speech production, and executive search processes (Chapman et al., 2020; Shao et al., 2014). These tasks may therefore provide a more direct measure of semantic representations and associative processing in semantic memory. One of the notable examples is the Québec Semantic Association Test (TASQ) (Macoir and Hudon, submitted) that was developed as a short measure of semantic associative processing in the forced‐choice paradigm. In each trial, participants choose the word that has the strongest meaningful association with a target stimulus, thus emphasizing conceptual relations over expressive language skills. The major strength of this approach is that it combines abstract and concrete concepts, which allows for the investigation of semantic processing across different conceptual domains. Semantic association paradigms might provide a clinically feasible and theoretically informative means of assessing semantic functioning by attenuating the influence of speech production and lexical retrieval demands, while at the same time sampling different semantic categories.

The development of a semantic association test for Turkish is particularly important because of the linguistic and cultural factors affecting semantic processing (Thompson et al., 2020). Turkish differs from many Indo‐European languages in its agglutinative morphology, lexical organization, and semantic structure (Göksel and Kerslake, 2005). Furthermore, semantic associations may be different across cultures as a function of language use, cultural experience, and the salience of concepts (Jonauskaite et al., 2025). As a result, the semantic relations formed in one linguistic or cultural context cannot be expected to generalize directly to another. While there are several measures with semantic components available to Turkish clinicians and researchers, such as the Turkish versions of the PPTT (Bozdemir and Gürvit, 2022), DTLA‐Tr (Karalı et al., 2025), and BECLA‐Tr (Karali et al., 2026), there is no brief measure specifically designed to assess semantic associative processing in both abstract and concrete conceptual domains. The lack of such a measure restricts the comprehensive assessment of semantic functioning in Turkish‐speaking adults and older adults and highlights the need for a culturally and linguistically appropriate instrument specifically targeting semantic association abilities.

The present study therefore aimed to develop and validate the Turkish Semantic Association Test (SEMAT), a culturally and linguistically appropriate measure of semantic association abilities in middle‐aged and older Turkish‐speaking adults. The study was conducted in three stages. Study 1 focused on the development of the SEMAT, including stimulus construction, expert evaluation, and pilot testing. Study 2 examined the psychometric properties of the SEMAT, including internal consistency and convergent validity. Study 3 established normative data and examined the effects of demographic variables on SEMAT performance.

Based on previous research on semantic cognition and healthy aging, demographic variables, particularly age and educational attainment, were expected to influence SEMAT performance. The SEMAT was also expected to demonstrate good internal consistency and significant positive associations with established semantic measures, supporting its validity as a brief assessment of semantic associative processing for use in clinical and research settings.

## 2 Study 1. Development of the SEMAT

### 2.1 Method

#### 2.1.1 Test Design and Rationale

The SEMAT assesses access to semantic representations from written input using a forced‐choice semantic association paradigm. On each trial, a target word appears at the top of the screen (e.g., öfke [anger]) and must be matched to one of two response options presented at the bottom left and right of the screen: a semantically related word (e.g., hiddet [rage]) and a semantically unrelated distractor (e.g., kaygı [anxiety]). Participants are instructed to select the word that is most meaningfully associated with the target stimulus. The test is designed to evaluate semantic association abilities while minimizing lexical retrieval and speech production demands that may influence performance on traditional naming‐based tasks. By incorporating both abstract and concrete concepts, the SEMAT provides a comprehensive assessment of semantic representations across different conceptual domains.

#### 2.1.2 Stimulus Selection and Psycholinguistic Control

The development of the Turkish SEMAT followed a multistep procedure designed to ensure cultural appropriateness, psycholinguistic suitability, and semantic validity of the stimuli. Although the Québec Semantic Association Test (TASQ) served as the conceptual inspiration for the development of the instrument, the SEMAT was specifically designed for Turkish‐speaking populations and involved the construction of a new set of semantic associations rather than a direct translation of the original stimuli.

Initially, the research team generated a preliminary pool of Turkish semantic triplets reflecting the theoretical principles underlying semantic association judgments. To maximize the sensitivity and discriminatory potential of the instrument, a larger pool of stimuli was created than required for the final version of the test. The pilot version included 100 word triplets distributed across different semantic domains, including abstract concepts, biological concepts (animals and fruits/vegetables), and man‐made concepts.

For concrete concepts, semantic relationships were constructed on the basis of perceptual and non‐perceptual attributes. Perceptual relationships included shared physical characteristics such as overall shape or size, whereas non‐perceptual relationships reflected features such as habitat, geographical origin, or functional use. For abstract concepts, semantic relationships were primarily based on synonymy. Synonym relationships between target and associated words were determined using standardized definitions and synonym information obtained from the official dictionaries of the Turkish Language Association (Türk Dil Kurumu [TDK], n.d.). Lexical frequency values for all items were extracted from the Turkish National Corpus (Aksan et al., 2012), and psycholinguistic characteristics of candidate stimuli were examined during item development.

#### 2.1.3 Face Validity, Pilot Testing, and Refinement

##### 2.1.3.1 Pilot‐Study and Final Selection of the Turkish SEMAT Stimuli

The pilot test included 100‐word triplets distributed as follows across three semantic domains: Abstract concepts (*n* = 32; concepts not directly accessible through sensory experience, referring to emotions, psychological states, personality traits, or ideas); Biological concepts (*n* = 42; 23 animals, 19 fruits/vegetables), and Manmade concepts (*n* = 26; e.g., tools and clothing).

Lexical frequency values for all items were extracted from the Turkish National Corpus (Aksan et al., 2012). For abstract concepts, synonym relationships between stimulus and target words were determined using standardized definitions and synonym information obtained from the official dictionaries of the Turkish Language Association (Türk Dil Kurumu [TDK], n.d.). Abstract concepts (*n* = 16) showed a mean lexical frequency of 71.97 (SD = 189.59), whereas concrete concepts (*n* = 32) showed a mean lexical frequency of 7.24 (SD = 8.69). Despite this numerical difference, the effect was not statistically significant, Welch's *t* = 1.36, *p* = 0.19, likely reflecting the substantial variability observed within the abstract category. Detailed characteristics of the stimuli are presented in Table 1.

**TABLE 1:** Characteristics of the stimuli used in the TSAT.

Semantic category	N	Stimulus‐target relationship	Example
Abstract concepts	28	Synonymy	**Reputation**: Celebrity—encouragement
Concrete concepts: Biological	32	Perceptual attribute (16) Non‐perceptual attribute (16)	**Donkey**: Cow—goat **Bat**: Pigeon—owl
Concrete concepts: Manmade	16	Perceptual attribute (8) Non‐perceptual attribute (8)	**Comb**: Shovel—saw **Key**: Hinge—lock

To establish face validity, the preliminary version of the SEMAT was independently reviewed by one neurologist and four speech and language therapists with experience in neurogenic communication disorders. Experts evaluated the appropriateness, clarity, semantic relevance, cultural suitability, and difficulty level of the stimuli and instructions. Written feedback and item‐specific recommendations were collected from all reviewers. Following the independent evaluations, the research team reviewed the experts’ comments and reached a consensus regarding the inclusion, modification, or exclusion of individual items. Revisions were subsequently implemented prior to pilot administration. The final version of the SEMAT retained a balanced representation of abstract and concrete semantic concepts and was designed to maximize sensitivity to subtle semantic processing deficits in neurodegenerative populations.

For the 42 concrete triplets (biological and manmade concepts), the three words within each triplet belong to the same broad semantic category. Associations are determined by specific attributes, which may be perceptual (e.g., size for animals, overall shape for fruits, vegetables, and objects) or non‐perceptual (e.g., habitat for animals, geographical origin for fruits and vegetables, and functional use for manmade objects).

For abstract concepts, associations are based on synonymy. Both stimulus and target words correspond to true synonyms according to a standardized synonym dictionary (Official Dictionary of the Turkish Language Association), whereas distractors are semantically related but not synonymous.

Prior to initiating the normative study, a pilot administration was conducted with 68 cognitively healthy adults aged 55 to 69 years (M = 61.97, SD = 4.47), with 4 to 17 years of education (M = 9.94, SD = 4.03). All participants obtained Montreal Cognitive Assessment (Nasreddine et al., 2005) scores within the normal range (*M* = 27.8, *SD* = 1.23). The objective of this phase was to evaluate task feasibility, clarity of instructions, and adequacy of scoring procedures. Participants received standardized oral instructions (in Turkish) explaining that they would see three written words per trial and would need to associate the top word with one of the two options presented below, based on general semantic knowledge. They were explicitly instructed not to rely on superficial similarities (e.g., shared letters or phonological overlap). Five practice trials were administered, each illustrating a different type of semantic relationship (e.g., synonymy, perceptual similarity, shared category membership, or functional association), with systematic corrective feedback provided. For example, participants were informed that *duş* [shower] and *küvet* [bath] are associated because both are used for washing, whereas *aşk* [love] and *sevgi* [affection] are related through synonymy.

Pilot performance was moderate to high (M = 86.1/100, SD = 8.6; range = 58–100), indicating that the task demands were appropriate for cognitively healthy adults. No administration difficulties were reported, and both the instructions and scoring procedures were considered clear, well understood, and suitable for clinical use.

Following the pilot administration, a final item selection procedure was conducted to optimize the psychometric and clinical properties of the task. Item selection was primarily guided by response accuracy in cognitively healthy adults, with the goal of minimizing both extreme ceiling effects and potentially ambiguous or excessively difficult items, while preserving adequate variability in performance. Item‐level accuracy was calculated as the proportion of participants providing the correct response to each item. Items answered correctly by ≥95% of participants were considered to show near‐ceiling performance. The ≥95% criterion was not an absolute exclusion cutoff; rather, near‐ceiling items were preferentially excluded when alternative items were available within the corresponding semantic category. Similarly, no fixed lower cutoff was applied. Items showing comparatively low accuracy were individually reviewed for potential ambiguity or excessive difficulty. Item selection also took into account semantic‐category representation and conceptual diversity. The final set of 76 items had item‐level accuracy rates ranging from 61.8% to 95.6%.

Because one of the primary objectives of the project was to compare semantic processing of abstract and concrete concepts in MCI and AD, the final version retained the maximum feasible number of abstract items. The final stimulus set therefore included 28 abstract triplets and 48 concrete triplets. Concrete items were distributed across semantic subcategories, including biological and man‐made concepts as well as perceptual and non‐perceptual associative relationships. The final version was designed to maintain balanced semantic representation while maximizing sensitivity to subtle semantic processing difficulties expected in neurodegenerative populations. The characteristics of the stimuli included in the SEMAT are presented in Table 1.

For administration, the SEMAT stimuli were integrated into a Microsoft PowerPoint presentation. Each slide contained one semantic triplet, with the target word presented at the top center of the slide and the two response options displayed below on the left and right sides. Participants were asked to indicate verbally which of the two alternatives was most meaningfully associated with the target word. Progression through the slides was controlled by the examiner, allowing participants unlimited response time and ensuring standardized administration across individuals.

## 3 Study 2. Psychometric Properties of the SEMAT

The objective of Study 2 was to examine selected psychometric properties of the SEMAT by evaluating its internal consistency and convergent validity. Internal consistency was assessed to determine the extent to which the SEMAT items measure a common semantic associative processing construct. Convergent validity was examined by investigating the relationship between SEMAT performance and established measures of semantic processing derived from the Turkish adaptation of the Batterie d’Évaluation Cognitive du Langage (BECLA‐Tr).

### 3.1 Participants

The participant sample consisted of 365 cognitively healthy Turkish‐speaking adults aged between 53 and 82 years. Participants were recruited from community settings through convenience sampling. Inclusion criteria were: (a) being a native speaker of Turkish, (b) age 50 years or older, (c) absence of self‐reported neurological, psychiatric, or developmental disorders, and (d) the ability to read and understand written Turkish. Individuals with a history of stroke, traumatic brain injury, neurodegenerative disease, severe psychiatric illness, or uncorrected sensory impairments likely to interfere with test performance were excluded. All participants underwent cognitive screening using the Montreal Cognitive Assessment (MoCA; Nasreddine et al., 2005). Individuals scoring within the normal range according to Turkish normative recommendations were included in the study. Demographic characteristics and cognitive screening performance are presented in Table 2.

**TABLE 2:** Characteristics of the normative sample.

Characteristic	Value
Sample size, *n* Age (years) Education (years) Sex, *n* (%) − Women − Men Cognitive screening − MoCA‐Tr score (/30) − DTLA‐Tr Total score (/100) TSAT Total score (/76)	365 62.35 (4.85) [53‐82] 10.76 (4.05) [4‐23] 178 (48.8) 187 (51.2) 25.65 (2.88) [18‐30] 94.04 (6.22) [74‐100] 68.43 (6.29) [42‐76]

*Note*: Continuous variables are presented as mean (SD) [range]. Categorical variables are presented as n (%).

### 3.2 Measures and Procedure

The SEMAT materials, administration procedure, instructions, and scoring methods were identical to those described in Study 1 (pilot testing). To evaluate convergent validity, participants also completed three semantic subtests from the BECLA‐Tr. These tasks were selected because they assess semantic processing abilities closely related to those targeted by the SEMAT. A composite semantic score was calculated by averaging the z‐scores obtained on the three BECLA‐Tr semantic measures, providing a global index of semantic performance.

### 3.3 Statistical Analysis

Internal consistency of the SEMAT was assessed using Cronbach's alpha and McDonald's omega coefficients. Both indices were calculated using responses to the 76 SEMAT items. Values above 0.70 were considered indicative of acceptable internal consistency, whereas values above 0.80 were considered good.

Convergent validity was examined by calculating Spearman rank‐order correlations between the BECLA‐Tr semantic composite score and the SEMAT Total Score, as well as the Abstract Concepts, Biological Concepts, and Manmade Concepts subscale scores. Spearman correlations were used because SEMAT score distributions deviated from normality and showed ceiling effects.

At the item level, difficulty was expressed as the proportion of participants responding correctly to each item, and discrimination was assessed using corrected item–total correlations, calculated between each item and the total SEMAT score excluding that item.

## 4 Results

### 4.1 Internal Consistency

The SEMAT demonstrated excellent internal consistency. Cronbach's alpha for the 76‐item test was 0.88, whereas McDonald's omega was 0.92. These findings indicate that the SEMAT items measure a common underlying construct while maintaining sufficient variability to capture individual differences in semantic associative processing.

### 4.2 Convergent Validity

Convergent validity was examined by correlating SEMAT scores with the BECLA‐Tr semantic composite score. As expected, significant positive correlations were observed between the BECLA‐Tr semantic composite and all SEMAT measures.

The SEMAT Total Score showed a large positive correlation with the BECLA‐Tr semantic composite score (ρ = 0.54, p < 0.001). Significant correlations were also observed for the Abstract Concepts score (ρ = 0.47, p < 0.001), Biological Concepts score (*ρ* = 0.48, *p* < 0.001), and Manmade Concepts score (ρ = 0.41, p < 0.001).

These findings provide evidence of good convergent validity and indicate that better performance on the SEMAT is associated with better performance on established measures of semantic processing. At the same time, the magnitude of the correlations suggests that the SEMAT assesses semantic associative processing abilities that are related to, but not entirely redundant with, those measured by the BECLA‐Tr.

### 4.3 Item Level Analysis

Item‐level analyses showed generally high accuracy across the 76 SEMAT items, with difficulty indices (proportion correct) ranging from 0.732 to 0.978. Corrected item–total correlations were positive for all items and ranged from 0.126 to 0.389, indicating variable item discrimination. Lower discrimination coefficients tended to be associated with higher item accuracy, consistent with the restricted response variability associated with the ceiling effects observed in the normative sample. Complete item‐level statistics are presented in Supplementary Table S1.

## 5 Study 3. Normative Data

The objective of Study 3 was to establish normative data for the SEMAT in Turkish‐speaking adults and older adults.

### 5.1 Participants

The participants included in Study 3 were the same cognitively healthy adults who participated in the psychometric study described in Study 2. The sample consisted of 365 Turkish‐speaking adults aged 53 to 82 years. Demographic characteristics and cognitive screening performance are presented in Table 2.

### 5.2 Measures and Procedure

The SEMAT was administered according to the procedures described in Study 2. Total SEMAT scores were calculated out of 76, with three semantic subscales corresponding to abstract concepts (28 points), biological concepts (32 points), and manmade concepts (16 points).

Each SEMAT item is scored dichotomously, with 1 point awarded for a correct response and 0 points for an incorrect response. The total score is calculated by summing the correct responses across all 76 items and ranges from 0 to 76. Three subscale scores are also calculated: Abstract Concepts (28 items; range: 0–28), Biological Concepts (32 items; range: 0–32), and Manmade Concepts (16 items; range: 0–16). Higher total and subscale scores indicate better performance on the corresponding semantic association tasks. Practice items are not included in the scores. SEMAT scores should be interpreted with reference to the normative data and the individual's demographic background, particularly educational attainment. Total scores summarize performance across the full test, whereas subscale scores describe performance within each semantic domain. Because the subscales contain different numbers of items, their raw scores should not be compared directly.

Performance below normative expectations does not, in itself, establish the presence of a semantic impairment or a neurocognitive disorder. SEMAT results should be considered alongside the individual's clinical history and findings from other language and cognitive assessments (Lezak et al., 2012). The present study provides normative reference data from cognitively healthy adults; diagnostic accuracy and clinically validated diagnostic thresholds remain to be established in clinical populations.

Participants were assessed individually in a single session either in a quiet room in their home or in a private testing room at the research center. Prior to the experimental trials, five practice items were administered. Instructions were repeated as needed to ensure task comprehension before the beginning of the test. Once the experimental phase had started, participants completed the SEMAT without interruption. Total administration time, including practice items and instructions, typically ranged from approximately 15 to 20 min. Recruitment and data collection were conducted between April and June 2026.

The standardized administration and scoring instructions, the scoring sheet, and the complete set of SEMAT stimuli are provided in the Supplementary Material. These resources specify the test procedures and scoring rules and provide access to the full instrument.

### 5.3 Statistical Analysis

Statistical analyses were conducted using Jamovi (version 2.6.45). Descriptive statistics were calculated for all demographic, cognitive screening, and SEMAT variables. Continuous variables are reported as means, standard deviations, and ranges, whereas categorical variables are presented as frequencies and percentages.

Inspection of score distributions revealed substantial ceiling effects for the SEMAT Total Score and all subscale scores. Shapiro–Wilk tests indicated significant departures from normality (all *p* < 0.001), and several distributions showed marked negative skewness. Consequently, nonparametric statistical procedures were used for univariate analyses of demographic effects.

Associations between demographic variables and SEMAT performance were first examined descriptively using Spearman correlations and Mann–Whitney *U* tests, given the non‐normal distributions of the SEMAT scores. To determine the independent contribution of demographic characteristics to SEMAT performance, multiple linear regression models were subsequently fitted for the total score and each semantic subscale. Age, education (years), and sex were entered simultaneously as prespecified predictors in all models; no stepwise or significance‐based variable selection procedure was used.

Because SEMAT scores are bounded and showed substantial ceiling effects and marked negative skewness, the suitability of the linear regression models for deriving regression‐based normative scores was also evaluated by examining the distribution of model residuals. Residual distributions from the linear regression models showed substantial departures from normality. Accordingly, the regression analyses were used to determine whether demographic adjustment was warranted rather than to derive individual regression‐based normative scores.

Demographic variables showing a consistent independent association with SEMAT performance across the multivariable models were considered for normative adjustment. Based on these analyses, normative data were stratified by educational attainment (see Results). Normative percentiles were therefore stratified by educational attainment. Participants were classified into three education groups (≤8, 9–14, and ≥15 years), selected to reflect the historically relevant eight‐year threshold in the Turkish education system while maintaining adequate sample sizes across normative strata (*n* = 146, 121, and 98, respectively). Differences between education groups were examined using Kruskal–Wallis tests, followed, when appropriate, by Dunn tests with Holm adjustment. Percentile values were calculated separately for the SEMAT Total Score, Abstract Concepts, Biological Concepts, and Manmade Concepts scores. Effect sizes for Mann–Whitney and Kruskal–Wallis analyses were expressed as r and Kruskal–Wallis eta‐squared (η^2^H), respectively.

### 5.4 Results

The normative sample included 365 Turkish‐speaking adults covering a broad range of age and educational attainment and comprising a balanced distribution of men and women. Descriptive characteristics of the sample, including cognitive screening measures and SEMAT performance, are presented in Table 2.

Inspection of score distributions revealed substantial ceiling effects for the SEMAT Total Score and all subscale scores. Shapiro–Wilk tests indicated significant departures from normality (all p < 0.001), and several distributions showed marked negative skewness. These distributional characteristics supported the use of empirical percentile‐based normative data.

In the multiple regression analyses, education was the only demographic variable independently associated with all SEMAT outcomes. For the total score, the overall model explained 10.8% of the variance (*R^2^* = 0.108); education was a significant predictor (*B* = 0.49, p < 0.001), whereas age (*B* = −0.11, p = 0.089) and sex (*B* = −0.19, *p* = 0.766) were not. Similar findings were obtained for the Abstract (*R^2^* = 0.071; education: *B* = 0.15, p < 0.001), Biological (*R^2^* = 0.092; education: *B* = 0.26, p < 0.001), and Manmade (*R^2^* = 0.049; education: *B* = 0.09, p < 0.001) subscales. Age and sex were not significant independent predictors in any of these models (all p > 0.05). Thus, education was the only demographic variable showing a consistent independent association with SEMAT performance and was retained as the demographic variable for normative stratification.

To further examine the influence of education, participants were divided into three educational groups (≤ 8 years, 9–14 years, and ≥ 15 years of education). Kruskal–Wallis analyses revealed significant group differences for the SEMAT Total Score, *H(2)* = 40.30, p < .001, *η^2^H* = .106, as well as for the Abstract Concepts score, *H(2)* = 36.65, p < 0.001, *η^2^H* = 0.096; Biological Concepts score, *H(2)* = 30.89, p < 0.001, *η^2^H* = 0.080; and Manmade Concepts score, *H(2)* = 17.07, p < 0.001, *η^2^H* = 0.042. Post hoc Dunn tests with Holm correction indicated significant differences among all three educational groups for the SEMAT Total Score, Abstract Concepts score, and Biological Concepts score (all corrected p ≤ 0.007). For the Manmade Concepts score, only the comparison between participants with ≤ 8 years and ≥ 15 years of education remained significant after correction (p < 0.001).

Because educational attainment was the only demographic variable consistently associated with SEMAT performance, normative data were stratified according to education level. Percentile ranks for the SEMAT Total Score and the three semantic subscales are presented in Table 3. The eighth percentile was selected as a normative reference threshold for identifying performance below the expected range, consistent with established neuropsychological interpretive conventions (Guilmette et al., 2020). This threshold should not be interpreted as a clinically validated diagnostic cutoff. For the SEMAT Total Score, the eighth‐percentile threshold corresponded to scores of 54, 62, and 65 for participants with ≤ 8 years, 9–14 years, and ≥ 15 years of education, respectively. Similar education‐related differences were observed for the Abstract Concepts, Biological Concepts, and Manmade Concepts subscales.

**TABLE 3:** Percentile ranks for TSAT total and subscale scores according to educational attainment.

	TSAT Total score (0‐76)			Abstract concepts (0‐28)			Biological concepts (0‐32)			Manmade concepts (0‐16)		
Percentiles	≤ 8 years	9‐14 years	≥ 15 years	≤ 8 years	9‐14 years	≥ 15 years	≤ 8 years	9‐14 years	≥ 15 years	≤ 8 years	9‐14 years	≥ 15 years
1	50	52	59	16	18	21	18	19	20	8	9	11
2	51	54	61	19	19	22	19	21	21	9	10	12
5	53	60	63	21	22	24	20	23	24	10	11	13
**8 (cutoff)**	**54**	**62**	**65**	**22**	**23**	**25**	**21**	**24**	**25**	**11**	**11**	**13**
10	56	62	65	22	23	25	21	25	26	11	12	13
15	60	63	67	23	25	26	23	25	27	12	12	14
25	62	65	69	24	26	27	24	26	27	13	14	14
35	64	67	70	26	26	27	26	27	28	14	14	15
45	67	69	71	26	27	28	27	28	30	14	15	15
50	67	70	72	26	27	28	28	28	30	14	15	15
55	68	71	73	27	27	28	28	29	30	15	15	16
65	69	72	74	27	28	28	29	29	31	15	16	16
75	71	73	75	27	28	28	29	31	32	16	16	16
85	73	75	76	28	28	28	31	32	32	16	16	16
90	74	76	76	28	28	28	31	32	32	16	16	16
95	76	76	76	28	28	28	32	32	32	16	16	16
98	76	76	76	28	28	28	32	32	32	16	16	16
99	76	76	76	28	28	28	32	32	32	16	16	16

*Note*: Normative data are presented separately for participants with ≤ 8 years, 9–14 years, and ≥ 15 years of education. Percentile ranks were derived from the normative sample of 365 cognitively healthy Turkish‐speaking adults. The eighth percentile is recommended as the cutoff for clinically abnormal performance, whereas scores between the eighth and 15th percentiles may be considered borderline.

## 6 Discussion

The aim of the present study was to develop the Turkish SEMAT, to provide normative data for Turkish‐speaking adults and older adults, and to examine some psychometric properties of the test. The results provide preliminary evidence that the SEMAT is a brief, culturally appropriate, and psychometrically sound measure of semantic associative processing. During the development phase, expert review and pilot administration were used to establish face validity and feasibility of the test. Normative analyses found that the most important demographic variable affecting performance on the SEMAT was educational attainment, with age and sex having only minor effects. Moreover, the SEMAT demonstrated excellent internal consistency and strong positive associations with established measures of semantic processing, supporting convergent validity. Taken together, these results suggest that the SEMAT is a psychometrically sound and theoretically driven tool for the assessment of semantic associative processing in Turkish‐speaking adults and older adults.

One of the major contributions of the present study is to develop a brief semantic association measure for Turkish‐speaking adults and older adults. Unlike traditional naming or verbal fluency tasks, semantic association paradigms mainly rely on the recognition of meaningful relationships between concepts and thus impose lower demands on lexical retrieval, speech production, and strategic search processes (Caputi et al., 2016; Chapman et al., 2020). This feature is particularly useful in the assessment of older adults and patients with neurodegenerative diseases, since performance on language tasks can be affected not only by semantic impairment but also by deficits in executive function, word retrieval, or speech production (Noonan et al.,^2010^; Jefferies, 2013). The expert review process and pilot administration supported the clarity, cultural appropriateness, and feasibility of the SEMAT, suggesting that the semantic associations selected for the Turkish version were easily understood by cognitively healthy participants. Moreover, the absence of administration problems and the relatively high performance of the pilot suggest that the task demands were appropriate and that the forced‐choice format provides a convenient means of assessing semantic associative processing in clinical and research settings (Zannino et al., 2021).

One of the distinctive features of the SEMAT is its integration of abstract and concrete semantic concepts within a single assessment framework. Most current semantic measures are heavily biased towards concrete concepts, object knowledge, or visually grounded semantic relationships, leaving little room to investigate possible differences between conceptual domains (Della Rosa et al., 2014; Hoffman et al., 2013). By contrast, the SEMAT was created to sample semantic associations to abstract, biological, and manmade concepts, allowing for a broader sampling of semantic representations. This distinction is important theoretically, as current models of semantic cognition suggest that abstract and concrete concepts differ in the cognitive and neural mechanisms underlying their processing, as well as in terms of representational content (Kousta et al., 2011; Lambon Ralph et al., 2017). Concrete concepts are more likely to be accompanied by richer perceptual and sensory representations, whereas abstract concepts are more reliant on linguistic, contextual, and relational information (Vigliocco et al., 2014; Mancano and Papagno, 2023). The inclusion of more semantic domains might thus increase the sensitivity of the SEMAT to subtle differences in semantic functioning. It might thus be especially useful in future research on semantic deficits in neurodegenerative disorders, where various conceptual categories might be differentially vulnerable. This characteristic may prove particularly valuable in future studies examining semantic impairment in Alzheimer's disease, primary progressive aphasia, and other neurodegenerative conditions in which different conceptual domains may be differentially affected.

The normative study, notably, found educational attainment to be a significant predictor of performance on the SEMAT. Performance on the total score and all semantic subscales was consistently better in those with higher levels of education. Educational attainment was the only demographic variable to have a systematic effect on performance. This finding aligns with numerous studies that have shown that educational experience is associated with development and maintenance of semantic and language abilities throughout adulthood and older age (Verhaeghen and Poncelet, 2013; Spaan, 2015). More educated people are likely to have advantages in vocabulary knowledge, linguistic experience, and opportunities to learn and organize semantic information across the life span (Opdebeeck et al., 2016). Educational attainment has also been identified as a key component of cognitive reserve, which can facilitate more efficient access to semantic knowledge and help with performance on cognitively demanding language tasks (Stern, 2009). The current results thus support the creation of education‐adjusted normative data and the need to consider educational background when interpreting SEMAT performance in clinical and research settings.

Age was not significantly correlated with SEMAT performance, in contrast to educational attainment. Although aging has been linked to declines in a number of language and cognitive processes, such as the speed of lexical retrieval and efficiency of word finding, semantic knowledge itself is generally believed to be relatively resistant to the effects of healthy aging (Brickman and Stern, 2009; Burke and Shafto, 2008; Rönnlund et al., 2005). Previous studies have suggested that older adults may suffer from problems in accessing the semantic information while the semantic representations are relatively intact (Verhaeghen and Poncelet, 2013). Thus, the absence of a significant age effect in the present study might be due to the nature of the SEMAT, which is based mainly on the recognition of semantic relations rather than the active retrieval and production of lexical items. This task minimizes demands on speech production and strategic search processes and may therefore be especially sensitive to the integrity of semantic representations themselves, which appear to remain relatively stable across healthy aging, at least within the age range represented in the present sample. These findings lend further support to the notion that age‐related changes in language performance need not be attributed to damage to semantic knowledge but may be due to inefficiencies in accessing or manipulating the knowledge. However, the restricted age range of the present sample may also have reduced the likelihood of detecting subtle age‐related effects.

The psychometric results also support the utility of the SEMAT as a measure of semantic associative processing. The internal consistency of the instrument was excellent. Cronbach's alpha and McDonald's omega coefficients showed that the items measure a common underlying construct but also have enough variation to account for individual differences. Moreover, SEMAT performance was significantly positively correlated with the semantic composite score from the BECLA‐Tr, providing evidence for convergent validity. These results suggest that SEMAT assesses abilities closely related to established constructs of semantic processing without being completely redundant with existing semantic assessment tools. This pattern is desirable from a psychometric perspective because convergent validity requires meaningful associations with related constructs without implying complete overlap. Indeed, the moderate‐to‐strong magnitude of the observed correlations suggests that the SEMAT measures a different dimension of semantic functioning, namely, the ability to identify meaningful associative relationships between concepts. Taken together, these findings support the psychometric robustness of the SEMAT and provide preliminary evidence for its validity as a brief measure of semantic associative processing.

The results of this study have several important clinical implications. Semantic impairments are amongst the most common language‐related problems reported in neurodegenerative disorders such as Alzheimer's disease, mild cognitive impairment, and primary progressive aphasia (Adlam et al., 2006; Gorno‐Tempini et al., 2011; Venneri et al., 2018). There is therefore a need for brief and clinically viable instruments that can assess semantic functioning without requiring excessive speech output or lexical retrieval. The SEMAT meets this need with a short, easy‐to‐administer measure of semantic associative processing that can be completed in about 15–20 min. Although the present study did not evaluate diagnostic accuracy, the observed psychometric properties suggest that the SEMAT may be a promising candidate for future clinical validation studies. The forced‐choice format reduces the influence of naming problems and executive search demands and may be especially useful for persons who have difficulty finding words but relatively preserved comprehension abilities. In addition, the combination of abstract and concrete semantic concepts may enable a more complete evaluation of semantic functioning and may be of use in future research investigating differential patterns of semantic impairment across neurodegenerative conditions. Overall, these features suggest that the SEMAT may be a useful adjunct to the assessment batteries available to clinicians and researchers working with aging populations and those with acquired language and cognitive disorders.

Several limitations of the present study should be mentioned. First, the normative sample included only cognitively healthy Turkish‐speaking adults and older adults, and thus the results cannot yet be generalized to clinical populations with acquired language or cognitive impairments. Accordingly, the percentile‐based normative thresholds provided in the present study should not be interpreted as diagnostic cutoffs. Although the eighth percentile provides a conventional reference point for identifying performance below normative expectations, its sensitivity and specificity for detecting semantic impairment have not yet been established. Second, participants were recruited by convenience sampling, which may limit the representativeness of the normative sample despite its relatively large size. Third, the current study examined selected psychometric properties, and additional evidence of test–retest reliability and responsiveness to cognitive change over time would further enhance the psychometric and clinical utility of the instrument. Therefore, future research should investigate the performance of individuals with mild cognitive impairment, Alzheimer's disease, primary progressive aphasia, and other neurodegenerative diseases to establish the diagnostic value of the SEMAT through diagnostic accuracy analyses, including sensitivity, specificity, and receiver operating characteristic (ROC) analyses, and to determine empirically supported clinical cutoff scores. Future studies using larger and more heterogeneous samples, thereby providing greater variability in item performance, could also apply Item Response Theory approaches to further characterize item difficulty and discrimination and to examine individual item functioning across the SEMAT performance continuum. Longitudinal studies may provide further clarification as to whether semantic associative processing changes in the course of neurodegenerative diseases and whether different conceptual domains, especially abstract and concrete concepts, exhibit different vulnerabilities. Such studies would contribute to a more complete understanding of semantic functioning and would further establish the clinical relevance of the SEMAT.

## 7 Conclusion

In summary, the present study introduces the Turkish SEMAT as a brief, culturally appropriate, and psychometrically strong measure of semantic associative processing for Turkish‐speaking adults and older adults. The test was developed via a systematic adaptation and validation process, demonstrated excellent internal consistency, and showed evidence of convergent validity. The normative results further demonstrated the impact of educational attainment on semantic associative performance, warranting the development of education‐adjusted normative data. The SEMAT assessment framework enables the incorporation of both abstract and concrete concepts and provides, therefore, a more comprehensive assessment of semantic functioning than many existing semantic measures. The SEMAT in general fills an important gap in the evaluation of semantic associative processing in Turkish and provides a promising tool for future clinical and research applications in aging and neurodegenerative disorders.

## Author Contributions

All authors contributed equally to the conception and design of the study, data collection, data analysis, interpretation of the results, and manuscript preparation. All authors reviewed and approved the final version of the manuscript.

## Funding

The authors have nothing to report.

## Ethics Statement

This study received ethical approval from the Biruni University Non‐Interventional Research Ethics Committee (Protocol No. 2024‐BİAEK/20‐25).

## Consent

Written informed consent was obtained from all participants prior to their inclusion in the study.

## Conflicts of Interest

The authors declare no conflicts of interest.

## Supporting information




**Supplementary Material**: brb371833‐sup‐0001‐SuppMat.pdf

**Supplementary Material**: brb371833‐sup‐0002‐SuppMat.pdf

**Supplementary Material**: brb371833‐sup‐0003‐SuppMat.xlsx

## Data Availability

The datasets generated and/or analyzed during the current study are available from the corresponding author upon reasonable request.

## References

[brb371833-bib-0001] Adlam, A. L. R. , S. Bozeat , R. Arnold , P. Watson , and J. R. Hodges . 2006. “Semantic Knowledge in Mild Cognitive Impairment and Mild Alzheimer's Disease.” Cortex; A Journal Devoted to the Study of the Nervous System and Behavior 42, no. 5: 675–684. 10.1016/S0010-9452(08)70404-0. 16909626

[brb371833-bib-0002] Aksan, Y. , M. Aksan , A. Koltuksuz , et al. 2012. “Construction of the Turkish National Corpus (TNC).” In Proceedings of the Eighth International Conference on Language Resources and Evaluation (LREC′12) , 3223–3227. European Language Resources Association (ELRA). http://www.lrec‐conf.org/proceedings/lrec2012/pdf/991_Paper.pdf.

[brb371833-bib-0003] Binder, J. R. , and R. H. Desai . 2011. “The Neurobiology of Semantic Memory.” Trends in Cognitive Sciences 15, no. 11: 527–536. 10.1016/j.tics.2011.10.001. PMC3350748 22001867

[brb371833-bib-0004] Binder, J. R. , R. H. Desai , W. W. Graves , and L. L. Conant . 2009. “Where Is the Semantic System? A Critical Review and Meta‐Analysis of 120 Functional Neuroimaging Studies.” Cerebral Cortex 19, no. 12: 2767–2796. 10.1093/cercor/bhp055. PMC2774390 19329570

[brb371833-bib-0005] Bozdemir, M. , and I. H. Gurvit . 2022. “Normative Data for the Turkish Version of the Pyramids and Palm Trees Test.” Applied Neuropsychology: Adult 29, no. 6: 1571–1577. 10.1080/23279095.2021.1900187. 33789062

[brb371833-bib-0006] Brickman, A. M. , and Y. Stern . 2009. “Aging and Memory in Humans.” In Handbook of the Neuroscience of Aging edited by P. R. Hof and C. V. Mobbs , 243–248. Elsevier.

[brb371833-bib-0007] Burke, D. M. , and M. A. Shafto . 2008. “Language and Aging.” In The Handbook of Aging and Cognition, edited by F. I. M. Craik and T. A. Salthouse , 3rd ed., 373–443. Psychology Press.

[brb371833-bib-0008] Caputi, N. , D. Di Giacomo , F. Aloisio , and D. Passafiume . 2016. “Deterioration of Semantic Associative Relationships in Mild Cognitive Impairment and Alzheimer Disease.” Applied Neuropsychology: Adult 23, no. 3: 186–195. 10.1080/23279095.2015.1030020. PMC4819827 26508434

[brb371833-bib-0009] Chapman, C. A. , T. T. Rogers , L. Ralph , and A. M . 2020. “Evaluating the Distinction Between Semantic Knowledge and Semantic Control Using Evidence From Semantic Aphasia and Semantic Dementia.” Psychonomic Bulletin & Review 27, no. 1: 63–77. 10.3758/s13423-019-01706-6. 31993976

[brb371833-bib-0010] Corbett, F. , E. Jefferies , A. Burns , and M. A. L. Ralph . 2012. “Unpicking the Semantic Impairment in Alzheimer's Disease: Qualitative Changes With Disease Severity.” Behavioural Neurology 25, no. 1: 23–34. 10.3233/BEN-2012-0346. PMC5294266 22207420

[brb371833-bib-0011] Cosgrove, A. L. , R. E. Beaty , C. W. Sandberg , Y. N. Kenett , and M. T. Diaz . 2026. “Semantic Memory Network Resilience in Aging: the Role of Abstract and Semantically Diverse Words.” Cognition 267: 106339. 10.1016/j.cognition.2025.106339. PMC13124224 41086687

[brb371833-bib-0012] Cousins, K. A. Q. , C. York , L. Bauer , and M. Grossman . 2016. “Cognitive and Anatomic Double Dissociation in the Representation of Concrete and Abstract Words in Semantic Variant and Behavioral Variant Frontotemporal Degeneration.” Neuropsychologia 84: 244–251. 10.1016/j.neuropsychologia.2016.02.025. PMC4808428 26944874

[brb371833-bib-0013] Croot, K. , L. Nickels , F. Laurence , and M. Manning . 2009. “Impairment‐ and Activity/Participation‐Directed Interventions in Progressive Language Impairment: Clinical and Theoretical Issues.” Aphasiology 23, no. 2: 125–160. 10.1080/02687030801943179.

[brb371833-bib-0014] Crutch, S. J. , and E. K. Warrington . 2005. “Abstract and Concrete Concepts Have Structurally Different Representational Frameworks.” Brain 128, no. 3: 615–627. 10.1093/brain/awh349. 15548554

[brb371833-bib-0015] Della Rosa, P. A. , E. Catricalà , G. Vigliocco , and S. F. Cappa . 2014. “How to Assess Abstract Conceptual Knowledge: Construction, Standardization and Validation of a New Battery of Semantic Memory Tests.” Functional Neurology 29, no. 1: 47–55. 10.11138/FNeur/2014.29.1.047. PMC4172247 25014049

[brb371833-bib-0016] Göksel, A. , and C. Kerslake . 2005. Turkish: a Comprehensive Grammar. Routledge.

[brb371833-bib-0017] Gorno‐Tempini, M. L. , A. E. Hillis , S. Weintraub , et al. 2011. “Classification of Primary Progressive Aphasia and Its Variants.” Neurology 76, no. 11: 1006–1014. 10.1212/WNL.0b013e31821103e6. PMC3059138 21325651

[brb371833-bib-0018] Guilmette, T. J. , J. J. Sweet , N. Hebben , et al. 2020. “American Academy of Clinical Neuropsychology Consensus Conference Statement on Uniform Labeling of Performance Test Scores.” The Clinical Neuropsychologist 34, no. 3: 437–453. 10.1080/13854046.2020.1722244. 32037942

[brb371833-bib-0019] Higby, E. , D. Cahana‐Amitay , A. Vogel‐Eyny , A. Spiro , M. L. Albert , and L. K. Obler . 2019. “The Role of Executive Functions in Object‐ and Action‐Naming Among Older Adults.” Experimental Aging Research 45, no. 4: 306–330. 10.1080/0361073X.2019.1627492. PMC6677138 31216948

[brb371833-bib-0020] Hodges, J. R. , and K. Patterson . 2007. “Semantic Dementia: A Unique Clinicopathological Syndrome.” The Lancet Neurology 6, no. 11: 1004–1014. 10.1016/S1474-4422(07)70266-1. 17945154

[brb371833-bib-0021] Hoffman, P. , M. A. Lambon Ralph , and T. T. Rogers . 2013. “Semantic Diversity: a Measure of Semantic Ambiguity Based on Variability in the Contextual Usage of Words.” Behavior Research Methods 45, no. 3: 718–730. 10.3758/s13428-012-0278-x. 23239067

[brb371833-bib-0022] Howard, D. , and K. Patterson . 1992. Pyramids and Palm Trees: A Test of Semantic Access From Words and Pictures. Thames Valley Test Company.

[brb371833-bib-0023] Invernizzi, S. , A. Bodart , L. Lefebvre , and I. S. Loureiro . 2023. “The Role of Semantic Assessment in the Differential Diagnosis Between Late‐Life Depression and Alzheimer's Disease or Amnestic Mild Cognitive Impairment: Systematic Review and Meta‐Analysis.” European Journal of Ageing 20, no. 1: 34. 10.1007/s10433-023-00780-z. PMC10415247 37563432

[brb371833-bib-0024] Jefferies, E. 2013. “The Neural Basis of Semantic Cognition: Converging Evidence From Neuropsychology, Neuroimaging and TMS.” Cortex; A Journal Devoted to the Study of the Nervous System and Behavior 49, no. 3: 611–625. 10.1016/j.cortex.2012.10.008. 23260615

[brb371833-bib-0025] Jonauskaite, D. , D. Epicoco , M. B. Meziane , et al. 2025. “Free Association Database for a 62‐Word Dataset Including Emotion and Colour Terms in English, Estonian, French, German, Italian, Lithuanian, and Spanish: Data From 14 Countries.” Journal of Open Psychology Data 13, no. 1: 4. 10.5334/jopd.140. PMC12330808 40777561

[brb371833-bib-0026] Karalı, F. S. , E. İ. Eskioğlu , S. Tosun , N. Çınar , and J. Macoir . 2025. “Turkish Adaptation, Reliability, and Validity of the Detection Test for Language Impairments in Adults and the Aged.” Applied Neuropsychology: Adult 32, no. 6: 1662–1669. 10.1080/23279095.2023.2301393. 38241752

[brb371833-bib-0027] Karali, F. S. , S. Tosun , E. İ. Eskioğlu , N. Cinar , and J. Macoir . 2026. “Adaptation and Normative Data of the Cognitive Assessment Battery of Language (BECLA‐Tr) for the Turkish Adult Population.” Aphasiology 40, no. 3: 447–464. 10.1080/02687038.2025.2451661.

[brb371833-bib-0028] Korthauer, L. E. , W. C. Heindel , L. Y. Lai , G. Tremont , B. R. Ott , and E. K. Festa . 2020. “Impaired Integration of Object Feature Knowledge in Amnestic Mild Cognitive Impairment.” Neuropsychology 34, no. 6: 699–712. 10.1037/neu0000670. 32551739

[brb371833-bib-0029] Kousta, S. T. , G. Vigliocco , D. P. Vinson , M. Andrews , and E. Del Campo . 2011. “The Representation of Abstract Words: Why Emotion Matters.” Journal of Experimental Psychology: General 140, no. 1: 14–34. 10.1037/a0021446. 21171803

[brb371833-bib-0030] Lambon Ralph, M. A. , E. Jefferies , K. Patterson , and T. T. Rogers . 2017. “The Neural and Computational Bases of Semantic Cognition.” Nature Reviews Neuroscience 18, no. 1: 42–55. 10.1038/nrn.2016.150. 27881854

[brb371833-bib-0031] Lezak, M. D. , D. B. Howieson , E. D. Bigler , and D. Tranel . 2012. Neuropsychological Assessment. 5th ed. Oxford University Press.

[brb371833-bib-0032] Luzzatti, C. , I. Mauri , S. Castiglioni , et al. 2020. “Evaluating Semantic Knowledge Through a Semantic Association Task in Individuals With Dementia.” American Journal of Alzheimer's Disease & Other Dementias® 35: 1533317520917294. 10.1177/1533317520917294. PMC10623912 32308008

[brb371833-bib-0033] Macoir, J. , and C. Hudon . submitted. “Strategic and Lexical Retrieval Processes in Free Verb Fluency: The Influence of Age and Education.” Manuscript submitted for publication. 10.1080/23279095.2025.2564369 41043804

[brb371833-bib-0034] Macoir, J. , R. Laforce , and C. Hudon . 2024. “The Impact of Lexical‐Semantic Impairment on Spoken Verb Production in Individuals With Mild Cognitive Impairment.” Cognitive and Behavioral Neurology 37, no. 4: 183–193. 10.1097/WNN.0000000000000378. 39359049

[brb371833-bib-0035] Mancano, M. , and C. Papagno . 2023. “Concrete and Abstract Concepts in Primary Progressive Aphasia and Alzheimer's Disease: A Scoping Review.” Brain Sciences 13, no. 5: 765. 10.3390/brainsci13050765. PMC10216362 37239237

[brb371833-bib-0036] Martin, A. , and L. L. Chao . 2001. “Semantic Memory and the Brain: Structure and Processes.” Current Opinion in Neurobiology 11, no. 2: 194–201. 10.1016/S0959-4388(00)00196-3. 11301239

[brb371833-bib-0037] Mkrtychian, N. , E. Blagovechtchenski , D. Kurmakaeva , D. Gnedykh , S. Kostromina , and Y. Shtyrov . 2019. “Concrete vs. abstract Semantics: from Mental Representations to Functional Brain Mapping.” Frontiers in Human Neuroscience 13: 267. 10.3389/fnhum.2019.00267. PMC6687846 31427938

[brb371833-bib-0057] Nasreddine, Z. S. , N. A. Phillips , V. Bédirian , et al. 2005. “The Montreal Cognitive Assessment, MoCA: A Brief Screening Tool for Mild Cognitive Impairment.” Journal of the American Geriatrics Society 53, no. 4: 695–699. 10.1111/j.1532-5415.2005.53221.x. 15817019

[brb371833-bib-0058] Noonan, K. A. , E. Jefferies , F. Corbett , and M. A. Lambon Ralph . 2010. “Elucidating the Nature of Deregulated Semantic Cognition in Semantic Aphasia: Evidence for the Roles of Prefrontal and Temporo‐Parietal Cortices.” Journal of Cognitive Neuroscience 22, no. 7: 1597–1613. 10.1162/jocn.2009.21289. 19580383

[brb371833-bib-0038] Opdebeeck, C. , A. Martyr , and L. Clare . 2016. “Cognitive Reserve and Cognitive Function in Healthy Older People: A Meta‐Analysis.” Aging, Neuropsychology, and Cognition 23, no. 1: 40–60. 10.1080/13825585.2015.1041450. 25929288

[brb371833-bib-0039] Patterson, K. , P. J. Nestor , and T. T. Rogers . 2007. “Where Do You Know What You Know? The Representation of Semantic Knowledge in the Human Brain.” Nature Reviews Neuroscience 8, no. 12: 976–987. 10.1038/nrn2277. 18026167

[brb371833-bib-0041] Rönnlund, M. , L. Nyberg , L. Bäckman , and L.‐G. Nilsson . 2005. “Stability, Growth, and Decline in Adult Life Span Development of Declarative Memory: Cross‐Sectional and Longitudinal Data From a Population‐Based Study.” Psychology and Aging 20, no. 1: 3–18. 10.1037/0882-7974.20.1.3. 15769210

[brb371833-bib-0040] Reilly, J. , J. E. Peelle , A. M. Garcia , and S. J. Crutch . 2016. “Linking Somatic and Symbolic Representation in Semantic Memory: The Dynamic Multilevel Reactivation Framework.” Psychonomic Bulletin & Review 23, no. 4: 1002–1014. 10.3758/s13423-015-0824-5. PMC5156531 27294419

[brb371833-bib-0042] Shao, Z. , E. Janse , K. Visser , and A. S. Meyer . 2014. “What Do Verbal Fluency Tasks Measure? Predictors of Verbal Fluency Performance in Older Adults.” Frontiers in Psychology 5: 772. 10.3389/fpsyg.2014.00772. PMC4106453 25101034

[brb371833-bib-0043] Spaan, P. E. 2015. “Episodic and Semantic Memory Functioning in Very Old Age: Explanations From Executive Functioning and Processing Speed Theories.” Cogent Psychology 2, no. 1: 1109782. 10.1080/23311908.2015.1109782.

[brb371833-bib-0059] Stern, Y. 2009. “Cognitive Reserve.” Neuropsychologia 47, no. 10: 2015–2028. 10.1016/j.neuropsychologia.2009.03.004. PMC2739591 19467352

[brb371833-bib-0049] Türk Dil Kurumu . n.d. Güncel Türkçe sözlük. https://sozluk.gov.tr.

[brb371833-bib-0044] Thompson, B. , S. G. Roberts , and G. Lupyan . 2020. “Cultural Influences on Word Meanings Revealed Through Large‐Scale Semantic Alignment.” Nature Human Behaviour 4, no. 10: 1029–1038. 10.1038/s41562-020-0924-8. 32778801

[brb371833-bib-0045] Thye, M. , P. Hoffman , and D. Mirman . 2024. “The Neural Basis of Naturalistic Semantic and Social Cognition.” Scientific Reports 14, no. 1: 6796. 10.1038/s41598-024-56897-3. PMC10957894 38514738

[brb371833-bib-0046] Tosun, S. , F. S. Karalı , E. İ. Eskioğlu , N. Çınar , and J. Macoir . 2026a. “Linguistic Vulnerabilities in Mild Cognitive Impairment: Evidence From the DTLA‐Tr Screening Battery.” Cortex; A Journal Devoted to the Study of the Nervous System and Behavior 198: 182–190. 10.1016/j.cortex.2026.03.007. 41864190

[brb371833-bib-0047] Tosun, S. , F. S. Karali , E. İ. Eskioğlu , N. Çinar , and J. Macoir . 2026b. “Assessing Language Impairments Across the Neurodegeneration Continuum.” Alzheimer Disease & Associated Disorders 40, no. 1: 52–58. 10.1097/WAD.0000000000000715. 41773885

[brb371833-bib-0048] Tulving, E. 1972. “Episodic and Semantic Memory.” In Organization of Memory, edited by E. Tulving and W. Donaldson , 381–403. Academic Press.

[brb371833-bib-0050] Venneri, A. , C. Jahn‐Carta , M. De Marco , D. Quaranta , and C. Marra . 2018. “Diagnostic and Prognostic Role of Semantic Processing in Preclinical Alzheimer's Disease.” Biomarkers in Medicine 12, no. 6: 637–651. 10.2217/bmm-2017-0324. 29896968

[brb371833-bib-0051] Verhaegen, C. , and M. Poncelet . 2013. “Changes in Naming and Semantic Abilities With Aging From 50 to 90 Years.” Journal of the International Neuropsychological Society 19, no. 2: 119–126. 10.1017/S1355617712001178. 23237304

[brb371833-bib-0052] Vigliocco, G. , S. T. Kousta , P. A. Della Rosa , et al. 2014. “The Neural Representation of Abstract Words: The Role of Emotion.” Cerebral Cortex 24, no. 7: 1767–1777. 10.1093/cercor/bht025. 23408565

[brb371833-bib-0053] Vigliocco, G. , and D. P. Vinson . 2007. “Semantic Representation.” In The Oxford Handbook of Psycholinguistics edited by M. G. Gaskell , 195–215. Oxford University Press.

[brb371833-bib-0054] Villani, C. , A. Loia , and M. M. Bolognesi . 2024. “The Semantic Content of Concrete, Abstract, Specific, and Generic Concepts.” Language and Cognition 16, no. 4: 867–894. 10.1017/langcog.2023.64.

[brb371833-bib-0055] Wang, J. , J. A. Conder , D. N. Blitzer , and S. V. Shinkareva . 2010. “Neural Representation of Abstract and Concrete Concepts: A Meta‐Analysis of Neuroimaging Studies.” Human Brain Mapping 31, no. 10: 1459–1468. 10.1002/hbm.20950. PMC6870700 20108224

[brb371833-bib-0056] Zannino, G. D. , R. Perri , C. Marra , et al. 2021. “The Free Association Task: Proposal of a Clinical Tool for Detecting Differential Profiles of Semantic Impairment in Semantic Dementia and Alzheimer's Disease.” Medicina 57, no. 11: 1171. 10.3390/medicina5711117. PMC8622204 34833389

