# Supplementary material for: Development, Normative Data, and Psychometric Properties of the Turkish Semantic Association Test (SEMAT) for Middle‐Aged and Older Adults
Source: Brain Behav. 2026 Oct 7;16(10):e71833. doi: 10.1002/brb3.71833 (PMC13644552; doi:10.1002/brb3.71833)
Supplement: Supplementary file 1 [file BRB3-16-e71833-s002.pdf]

SEMANTIC ASSOCIATION TEST

Instructions: In this task, you will see three words at the same time. You will be asked to read the word at the top of the screen and match it with one of the two words at the bottom. You should choose the word that is most closely related to the top word based on your general knowledge. The relationship may involve physical characteristics of objects (such as shape or size), their functions, or the meanings of the words. However, you should not match words solely because they begin or end with the same letters or because they rhyme. A few practice examples are provided below to help you understand the task.

Note to the examiner: Provide feedback on every practice trial, regardless of whether the participant's response is correct or incorrect.

EXAMPLE 1

Abstract Words – LOVE: affection – kindness (Feedback: Synonymy)

EXAMPLE 2

Concrete Words – Animals: WASP: mosquito – bee (Feedback: Size)

EXAMPLE 3

Concrete Words – Plants: VIOLET: fern – rose (Feedback: Flowering plants)

EXAMPLE 4

Concrete Words – Manmade Objects: BIKE: car – motorcycle (Feedback: Two-wheeled vehicles)

EXAMPLE 5

Concrete Words – Manmade Objects: SHOWER: bathtub – pool (Feedback: Places used for bathing)

Item No.	TARGET WORD	OPTION 1	OPTION 2	TYPE	SCORE (1 OR 0)
1.	UNUTMA	karıştırma	**atlama**	A	
2.	ŞEFTALİ	**elma**	muz	D	
3.	İTİBAR	**şöhret**	cesaret	A	
4.	KORKMA	**ürkme**	şaşırma	A	
5.	DÜMEN	**direksiyon**	tencere	G	
6.	MERAK	**ilgi**	kayıtsızlık	A	
7.	KARTAL	**şahin**	akbaba	B	
8.	HEVES	**istek**	sabırsızlık	A	
9.	ONAY	**izin**	yönerge	A	
10.	KUŞKONMAZ	patates	**havuç**	E	
11.	ÖZGÜVEN	**cesaret**	çekingenlik	A	
12.	KEP	bere	**şapka**	F	
13.	İSTEK	dua	**dilek**	A	
14.	KOPYA	açıklama	**taklit**	A	
15.	EŞEK	**inek**	keçi	C	
16.	DOMATES	havuç	**biber**	D	
17.	ÖZLEM	yabancılık	**hasret**	A	
18.	KIZAK	paten	**kayak**	G	
19.	KIRLANGIÇ	karga	**serçe**	C	
20.	ZEBRA	**ceylan**	fil	C	
21.	ENGİNAR	**kereviz**	ananas	E	
22.	CEZA	**yaptırım**	öğrenme	A	
23.	DEĞİRMEN	roket	**helikopter**	F	
24.	YARASA	güvercin	**baykuş**	B	
25.	ANANAS	**kayısı**	elma	D	
26.	ENDİŞE	sıkıntı	**kaygı**	A	
27.	PANİK	kurtarma	**telaş**	A	
28.	KOYUN	**at**	tilki	B	
29.	PENSE	**makas**	testere	F	
30.	DESTEK	onay	**yardım**	A	
31.	TESTERE	**balta**	matkap	G	
32.	ANAHTAR	menteşe	**kilit**	G	
33.	DİKKAT	dağınıklık	**odak**	A	
34.	SİGARA	**kalem**	top	F	
35.	ÇAM	palmiye	**ladin**	D	
36.	HAYRANLIK	küçümseme	**beğeni**	A	
37.	KABAK	bezelye	**pırasa**	E	
38.	KARMAŞA	**bozulma**	şaşırma	A	
39.	ÜZÜNTÜ	**keder**	kin	A	
40.	ÖRDEK	**kuğu**	tavuk	B	
41.	İTİRAF	samimiyet	**ifşa**	A	
42.	KÜLLÜK	**tabak**	vazo	F	
43.	BAŞARI	**kazanım**	cesaret	A	
44.	GEYİK	tavşan	**ayı**	C	
45.	BALKABAĞI	**karpuz**	elma	E	
46.	TARAK	kürek	**testere**	F	
47.	KEMER	**askı**	ayakkabı	G	
48.	MAYMUN	**aslan**	kokarca	B	
49.	AHUDUDU	**böğürtlen**	üzüm	D	
50.	KARARLILIK	coşku	**azim**	A	
51.	ALARM	korna	**siren**	G	

52.	ASLAN	**kaplan**	eşek	C	
53.	KAYGI	**tedirginlik**	rahatlık	A	
54.	KİRAZ	muz	**vişne**	D	
55.	ÖZVERİ	bencillik	**yardımseverlik**	A	
56.	HAVUÇ	**turp**	şeftali	E	
57.	HUZURSUZLUK	**tedirginlik**	şaşkınlık	A	
58.	PENSE	**kerpeten**	tornavida	F	
59.	GÜVEN	**sadakat**	korku	A	
60.	BAVUL	**valiz**	çanta	G	
61.	PİŞMANLIK	**vicdan**	öfke	A	
62.	PROJEKTÖR	**ekran**	radyo	G	
63.	MERHAMET	**şefkat**	özlem	A	
64.	KAYIP	**yas**	umut	A	
65.	PELİKAN	**martı**	devekuşu	B	
66.	SIRTLAN	**kurt**	geyik	B	
67.	BARBUNYA	**fasulye**	salatalık	E	
68.	YUNUS	köpekbalığı	**balina**	B	
69.	ERİK	**kayısı**	kavun	D	
70.	DEVE	**lama**	keçi	C	
71.	LİMON	**greyfurt**	elma	D	
72.	KEREVİZ	**pırasa**	patates	E	
73.	KARİDES	**yengeç**	midye	C	
74.	BÖĞÜRTLEN	**dut**	üzüm	D	
75.	KURT	**çakal**	tilki	B	
76.	DÜRBÜN	**teleskop**	mikroskop	G	
