# Supplementary material for: Development, Normative Data, and Psychometric Properties of the Turkish Semantic Association Test (SEMAT) for Middle‐Aged and Older Adults
Source: Brain Behav. 2026 Oct 7;16(10):e71833. doi: 10.1002/brb3.71833 (PMC13644552; doi:10.1002/brb3.71833)
Supplement: Supplementary file 2 [file BRB3-16-e71833-s003.pdf]

Semantic Association Test

SEMAT

In this task, you will see three words at the same time. You will be asked to read the word at the top of the screen and match it with one of the two words at the bottom. You should choose the word that is most closely related to the top word based on your general knowledge. The relationship may involve the physical characteristics of objects (such as shape or size), their functions, or the meanings of the words. However, you should not match words solely because they begin or end with the same letters or because they rhyme. A few examples are provided below to help you understand the task.

Example

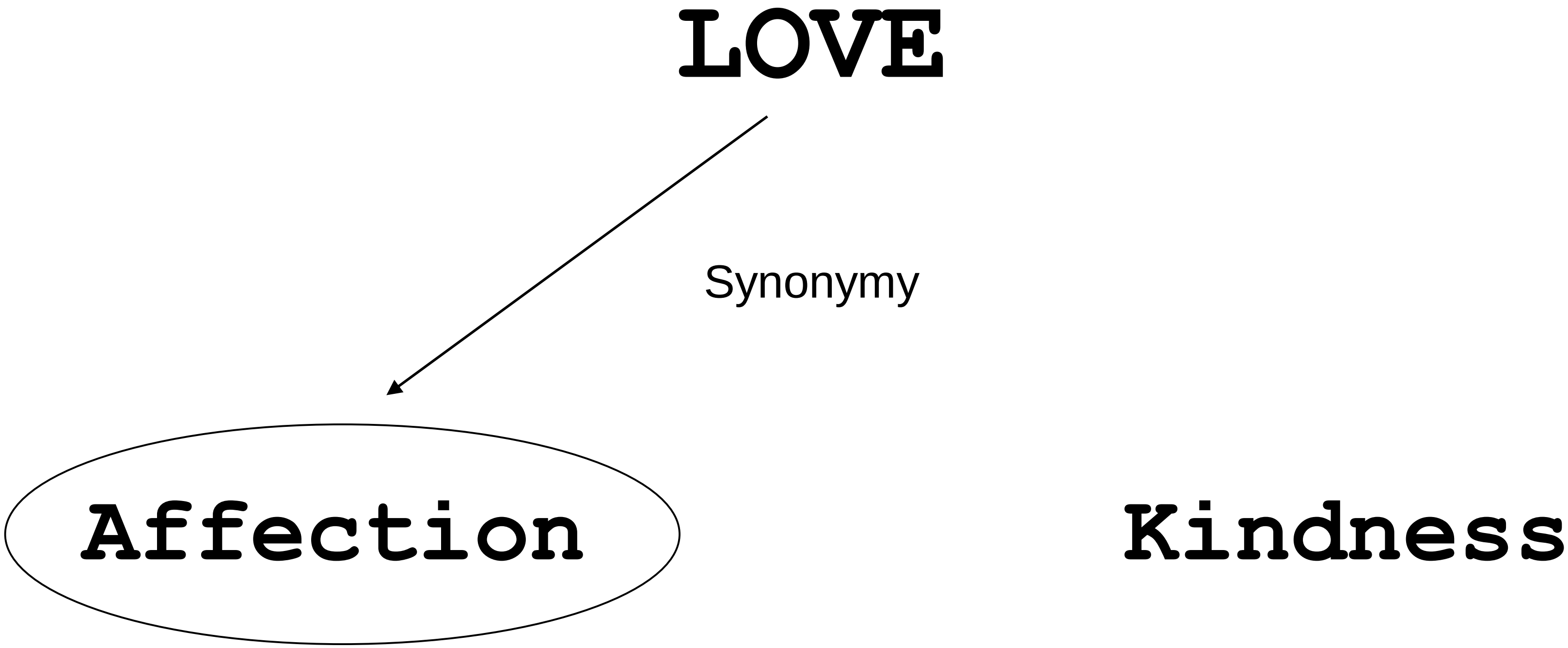

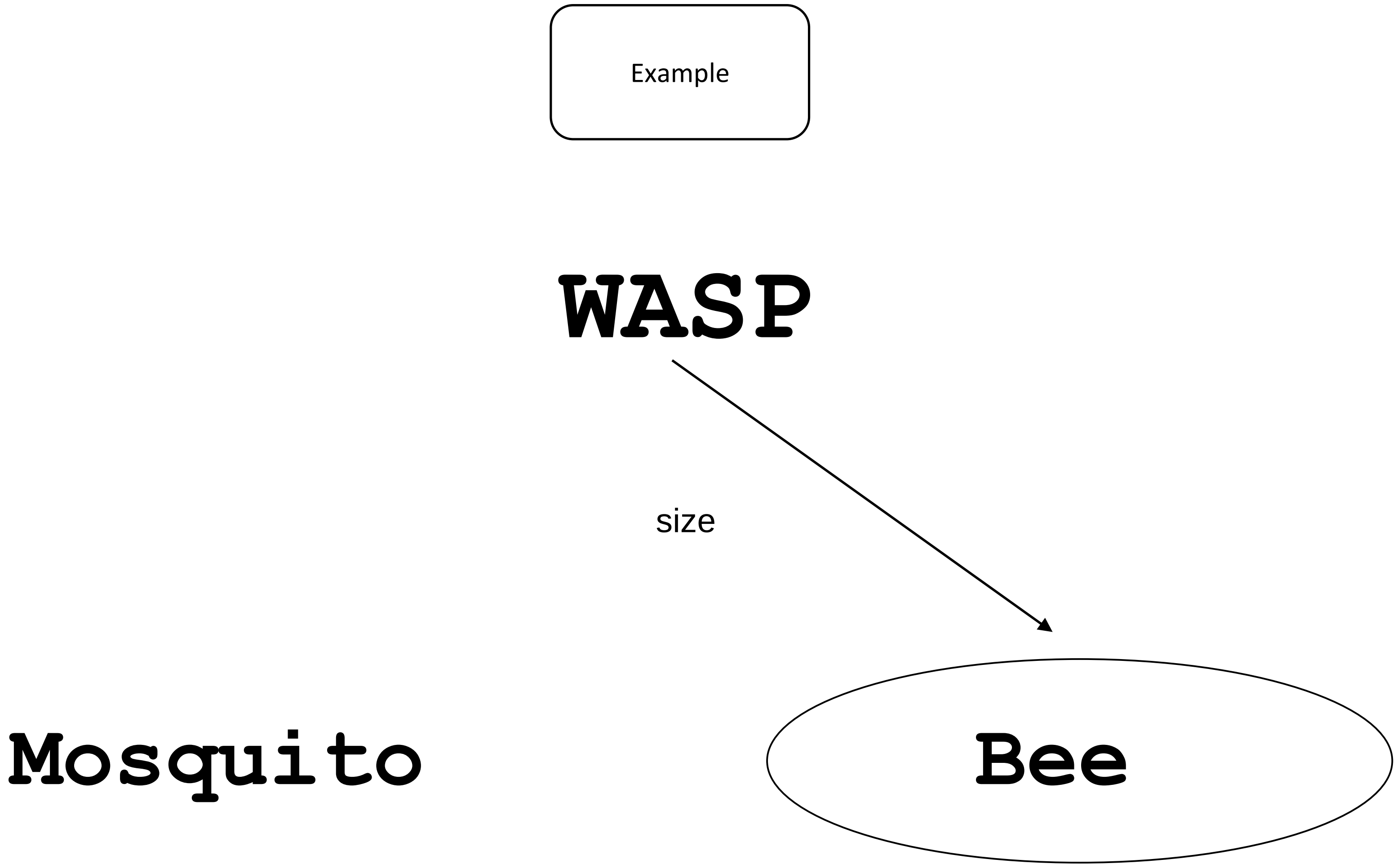
Example
WASP
size
Mosquito
Bee

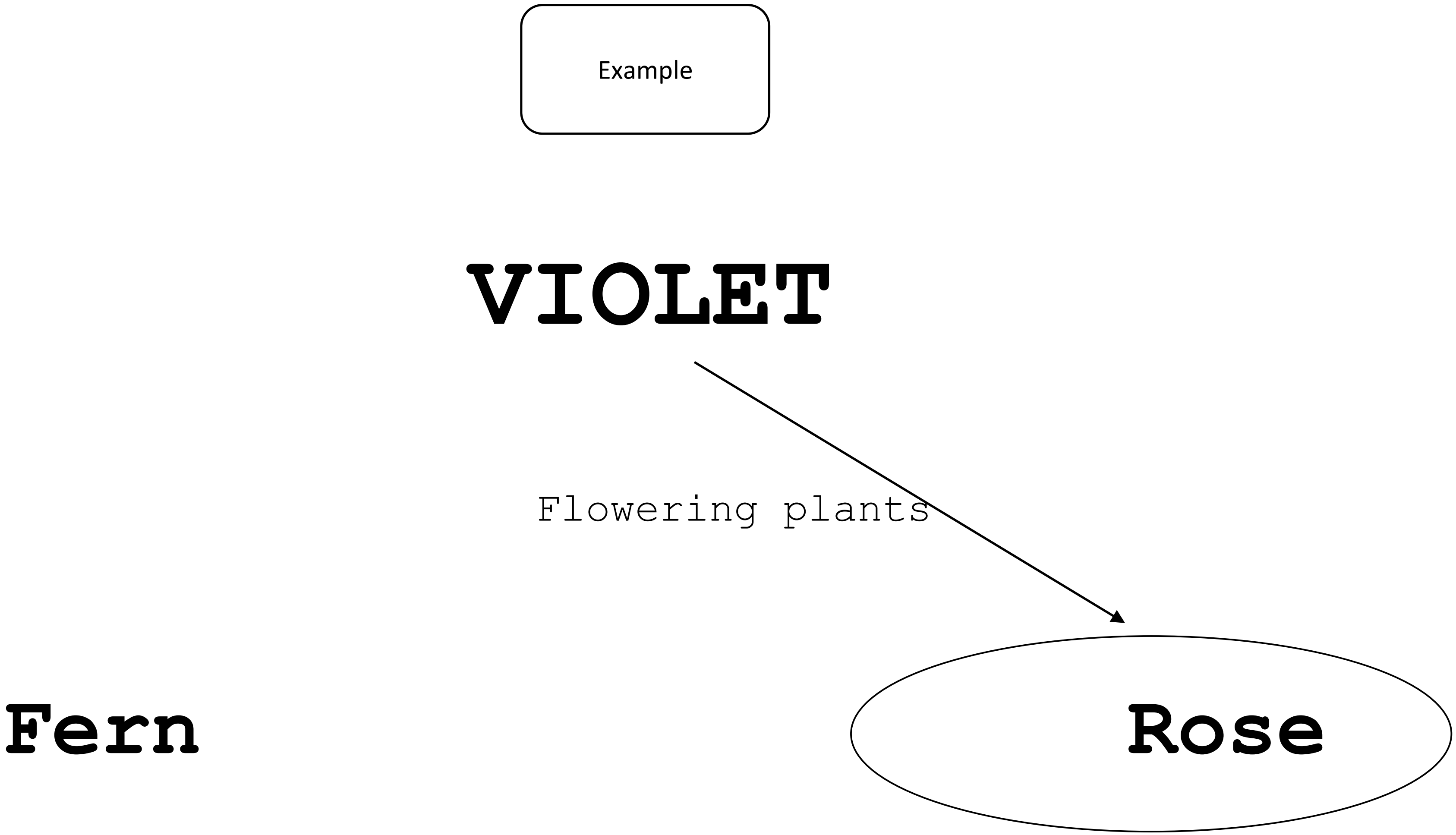
Example
VIOLET
Flowering plants
Fern
Rose

Example

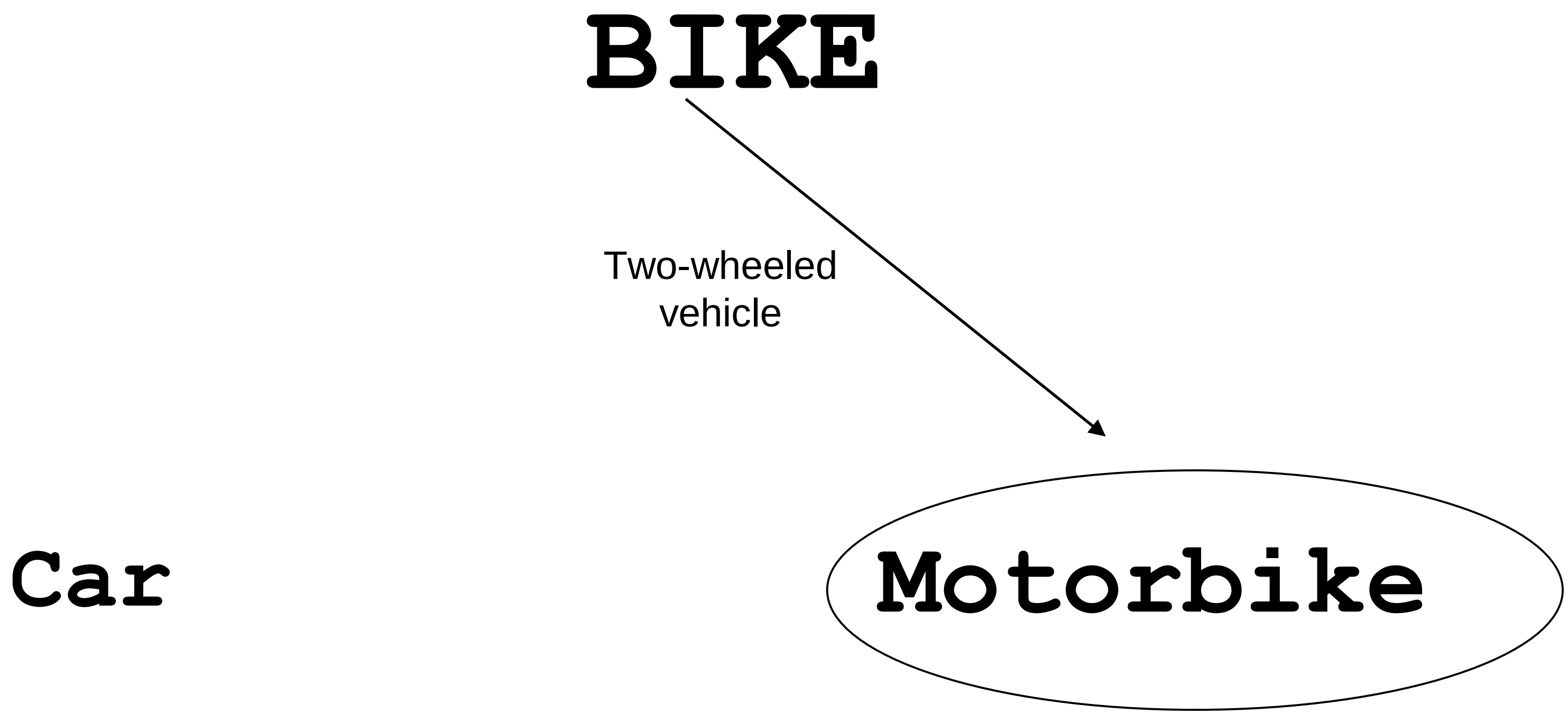

Example

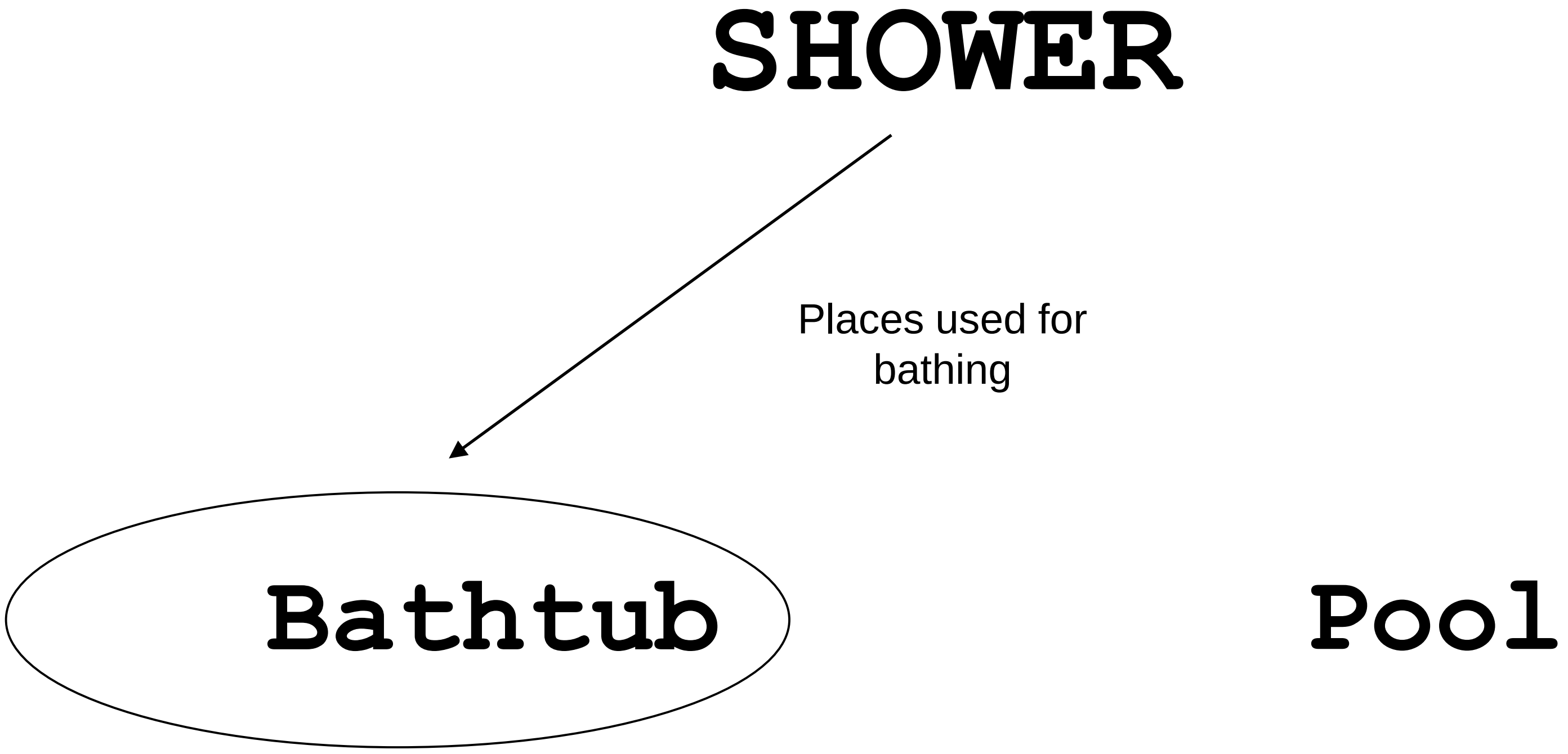

LET'S BEGIN THE TEST!

UNUTMA

karıştırma **atlama**

ŞEFTALİ

elma

muz

İTİBAR

şöhret

cesaret

KORKMA

ürkme şaşırma

DÜMEN

direksiyon tencere

MERAK

ilgi

kayıtsızlık

KARTAL

şahin

akbaba

HEVES

istek

sabırsızlık

ONAY

izin

yönerge

KUŞKONMAZ

patates havuç

ÖZGÜVEN

cesaret **çekingenlik**

KEP

bere **şapka**

İSTEK

dua

dilek

KOPYA

açıklama taklit

EŞEK

inek

keçi

DOMATES

havuç

biber

ÖZLEM

yabancılık **hasret**

KIZAK

paten　　　　kayak

KIRLANGIÇ

karga serçe

ZEBRA

ceylan **fil**

ENGİNAR

kereviz

ananas

CEZA

yaptırım öğrenme

DEĞİRMEN

roket helikopter

YARASA

güvercin baykuş

ANANAS

kayısı

elma

ENDİŞE

sıkıntı kaygı

PANİK

kurtarma telaş

KOYUN

at tilki

PENSE

makas testere

DESTEK

onay

yardım

TESTERE

balta matkap

ANAHTAR

menteşe kilit

DİKKAT

dağınıklık   odak

SİGARA

kalem top

ÇAM

palmiye ladin

HAYRANLIK

küçümseme beğeni

KABAK

bezelye pırasa

KARMAŞA

bozulma şaşırma

ÜZÜNTÜ

keder

kin

ÖRDEK

kuğu

tavuk

İTİRAF

samimiyet **ifşa**

KÜLLÜK

tabak vazo

BAŞARI

kazanım

cesaret

GEYİK

tavşan

ayı

BALKABAĞI

karpuz **elma**

TARAK

kürek testere

KEMER

askı

ayakkabı

MAYMUN

aslan kokarca

AHUDUDU

böğürtlen üzüm

KARARLILIK

coşku **azim**

ALARM

korna **siren**

ASLAN

kaplan **eşek**

KAYGI

tedirginlik rahatlık

KİRAZ

muz

vişne

ÖZVERİ

bencilllik

yardımseverlik

HAVUÇ

turp

şeftali

HUZURSUZLUK

tedirginlik

şaşkınlık

PENSE

kerpeten tornavida

GÜVEN

sadakat

korku

BAVUL

valiz

çanta

PİŞMANLIK

vicdan **öfke**

PROJEKTÖR

ekran **radyo**

MERHAMET

şefkat

özlem

KAYIP

yas

umut

PELİKAN

martı

devekuşu

SIRTLAN

kurt

geyik

BARBUNYA

fasülye **salatalık**

YUNUS

köpekbalığı

balina

ERİK

kayısı

kavun

DEVE

lama

keçi

LİMON

greyfurt **elma**

KEREVİZ

pırasa

patates

KARİDES

yengeç

midye

BÜĞÜRTLEN

dut

üzüm

KURT

çakal

tilki

DÜRBÜN

teleskop

mikroskop

The test is now complete.

Thank you for your participation!
